# Systemic Biomarkers of Neutrophilic Inflammation, Tissue Injury and Repair in COPD Patients with Differing Levels of Disease Severity

**DOI:** 10.1371/journal.pone.0038629

**Published:** 2012-06-12

**Authors:** Debra A. Cockayne, Donavan T. Cheng, Benjamin Waschki, Sriram Sridhar, Palanikumar Ravindran, Holly Hilton, Galina Kourteva, Hans Bitter, Sreekumar G. Pillai, Sudha Visvanathan, Kai-Christian Müller, Olaf Holz, Helgo Magnussen, Henrik Watz, Jay S. Fine

**Affiliations:** 1 Inflammation Disease Therapy Area, Hoffmann-La Roche, Nutley, New Jersey, United States of America; 2 Translational Research Sciences, Hoffmann-La Roche, Nutley, New Jersey, United States of America; 3 Pulmonary Research Institute at Hospital Grosshansdorf, Grosshansdorf, Germany; 4 Center for Pneumology and Thoracic Surgery, Hospital Grosshandorf, Grosshansdorf, Germany; University of Rochester Medical Center, United States of America

## Abstract

The identification and validation of biomarkers to support the assessment of novel therapeutics for COPD continues to be an important area of research. The aim of the current study was to identify systemic protein biomarkers correlated with measures of COPD severity, as well as specific protein signatures associated with comorbidities such as metabolic syndrome. 142 protein analytes were measured in serum of 140 patients with stable COPD, 15 smokers without COPD and 30 non-smoking controls. Seven analytes (sRAGE, EN-RAGE, NGAL, Fibrinogen, MPO, TGF-α and HB-EGF) showed significant differences between severe/very severe COPD, mild/moderate COPD, smoking and non-smoking control groups. Within the COPD subjects, univariate and multivariate analyses identified analytes significantly associated with FEV_1_, FEV_1_/FVC and DLCO. Most notably, a set of 5 analytes (HB-EGF, Fibrinogen, MCP-4, sRAGE and Sortilin) predicted 21% of the variability in DLCO values. To determine common functions/pathways, analytes were clustered in a correlation network by similarity of expression profile. While analytes related to neutrophil function (EN-RAGE, NGAL, MPO) grouped together to form a cluster associated with FEV_1_ related parameters, analytes related to the EGFR pathway (HB-EGF, TGF-α) formed another cluster associated with both DLCO and FEV_1_ related parameters. Associations of Fibrinogen with DLCO and MPO with FEV_1_/FVC were stronger in patients without metabolic syndrome (*r*  =  −0.52, *p*  = 0.005 and *r*  =  −0.61, *p*  = 0.023, respectively) compared to patients with coexisting metabolic syndrome (*r*  =  −0.25, *p*  = 0.47 and *r  = * −0.15, *p*  = 0.96, respectively), and may be driving overall associations in the general cohort. In summary, our study has identified known and novel serum protein biomarkers and has demonstrated specific associations with COPD disease severity, FEV_1_, FEV_1_/FVC and DLCO. These data highlight systemic inflammatory pathways, neutrophil activation and epithelial tissue injury/repair processes as key pathways associated with COPD.

## Introduction

Chronic obstructive pulmonary disease (COPD) is a heterogeneous condition variably characterized by progressive airflow limitation with dyspnea, airway inflammation and remodeling, lung parenchymal destruction with emphysema, systemic inflammation and comorbidities including cardiovascular disease and metabolic syndrome [1], [2]. Currently, there are limited therapies to prevent disease progression, effectively treat inflammation or reduce mortality, and the development of novel treatment strategies will require a deeper understanding of the underlying inflammatory and tissue injury/repair processes associated with COPD disease pathogenesis [3]. Moreover, while therapeutic trials have largely relied on FEV_1_ as a primary endpoint and the central measure of disease severity, FEV_1_ remains a poor surrogate for disease activity and for distinguishing COPD sub-phenotypes [4], [5], [6]. Thus, there is a need for surrogate biomarkers, including accessible blood biomarkers, to support COPD patient stratification for clinical trials or to serve as predictors of disease progression, extrapulmonary effects and comorbidities [5], [6].

Despite intensive research only a few disease-related markers have emerged. C-reactive protein (CRP), Fibrinogen and IL-6 are reported to be related to systemic inflammation [7], [8], [9], [10], exacerbations and other lung function parameters [11], [12] in COPD. Elevated levels of soluble TNF receptor-1 (sTNFR-1), Osteoprotegerin and neutrophil gelatinase associated lipocalin (NGAL) were associated with GOLD stage and frequency of exacerbation in the Bergen COPD cohort [13], [14], and myeloperoxidase (MPO) and vascular endothelial growth factor (VEGF) were associated with the severity of lung function impairment and dyspnea in a separate study [15]. Several studies, including those analyzing the ECLIPSE COPD cohort [16], [17], [18], [19], [20], have identified decreased circulating levels of the Clara cell protein CC-16 in patients with COPD, suggesting that this protein may be a marker of potential interest in predicting bronchial epithelial cell dysfunction. In a multiplex profiling study, Pinto-Plata et al. [8] identified a panel of 24 biomarkers in 48 patients with COPD that showed associations with FEV_1_ and the BODE index, including proteins associated with inflammation, chemoattraction and tissue destruction.

The Grosshansdorf COPD cohort has been instrumental in defining novel associations between systemic inflammation and extrapulmonary comorbidities such as metabolic syndrome [21], [22], [23], [24], [25], [26], [27]. In the current study, we extended our assessment of this cohort by performing a cross-sectional analysis of 142 systemic biomarkers in COPD patients, smokers without COPD and healthy non-smoking controls. In selecting the protein analytes to be interrogated in this study we focused first on known biomarkers in COPD, and expanded this panel to include a wider array of protein analytes associated with biological pathways hypothesized to play a role in COPD disease pathogenesis, including inflammation and chemokine signaling, growth factor/tissue repair pathways and metabolic regulation. We performed analyses in this study to address three objectives. The first objective was to identify systemic biomarker changes associated with COPD disease severity, comparing COPD patients against smoking and non-smoking control subjects. The second objective was to identify associations between biomarkers and lung function parameters in COPD patients using univariate and multivariate regression. Lastly, metabolic syndrome has been observed to be a frequent comorbidity in patients with COPD, and previous studies by Watz et al. [21] found increased levels of IL-6 and hs-CRP in patients with COPD and coexisting metabolic syndrome compared to COPD patients without metabolic syndrome. Thus, the third objective was to determine if biomarkers associated with lung function parameters differed in COPD patients with or without coexisting metabolic syndrome. The identified associations may contribute toward a better overall understanding of COPD pathogenesis and inform future biomarker studies in COPD.

## Results

### Clinical Characteristics of Cohort

The Grosshansdorf COPD cohort used in the present study consisted of a total of 185 subjects including 30 healthy non-smokers, 15 smoking controls with chronic bronchitis (i.e. GOLD stage 0 in former GOLD classification scheme, on average 43 pack years of smoking) and 140 patients with COPD (GOLD stage I-IV). Of these 140 COPD subjects, 65 had metabolic syndrome. The baseline characteristics of the 185 subjects, including demographics, smoking history, steroid use and clinical measurements are summarized in Table 1.

**Table 1:** Characteristics of study cohort.

	Total	Non-Smokers	Smokers	GOLD I/II	GOLD III/IV	*p*
Patients (*n*)	185 (100%)	30 (16%)	15 (8%)	75 (41%)	65 (35%)	
***Demographics***						
Age in years	66.6 (0.5)	66.4 (1.1)	66.8 (1.7)	66.9 (0.7)	66.3 (0.7)	0.94
Males (*n*)	135 (73%)	21 (70%)	11 (73%)	54 (72%)	49 (75%)	0.95
Body Mass Index (BMI)	26.0 (0.4)	26.0 (0.8)	26.3 (0.8)	27.3 (0.5)	24.3 (0.7)	<0.001
Pack years	45.3 (2.2)	0.5 (0.2)	42.9 (5.3)	51.9 (2.9)	58.8 (3.1)	<0.001
Current smokers (*n*)	59 (32%)	0 (0%)	10 (67%)	30 (40%)	19 (29%)	<0.001
Systemic steroid use *(n*)	25 (14%)	0 (0%)	0 (0%)	5 (2.7%)	20 (11%)	<0.001
Theophylline use *(n*)	20 (11%)	0 (0%)	0 (0%)	2 (1.1%)	18 (9.7%)	<0.001
***Metabolic*** ** ***Syndrome***						
Metabolic syndrome *(n*)	88 (48%)	13 (43%)	10 (67%)	44 (59%)	21 (32%)	0.006
***Lung*** ** ***Function*** ** ***Parameters***						
Post bronchodilator FEV_1%_ predicted	65.0 (43.0 – 96.0)	114.0 (108.5 −125.0)	100.0 (92.5 − 107.0)	75.0 (62.0 − 85.0)	36.0 (27.0 − 45.0)	<0.001
Post bronchodilator FEV_1_/FVC ratio	56.1 (43.8 − 72.8)	78.3 (75.8 − 80.9)	76.7 (74.8 − 78.7)	60.2 (51.2 − 65.8)	39.1 (34.0 − 46.0)	<0.001
DLCO % predicted (Hb corrected)	60.1 (39.5 − 74.8)	80.2 (74.8 − 89.1)	70.9 (61.5 − 81.6)	61.0 (51.7 − 73.0)	31.6 (24.8 − 42.1)	<0.001

Data are expressed as the number of subjects (% of subjects), mean (SEM) or median (Interquartile range) for lung function parameters. COPD subjects were grouped as GOLD I/II (mild/moderate) and GOLD III/IV (severe/very severe).

### Overview of Study Analysis

**Figure 1:**
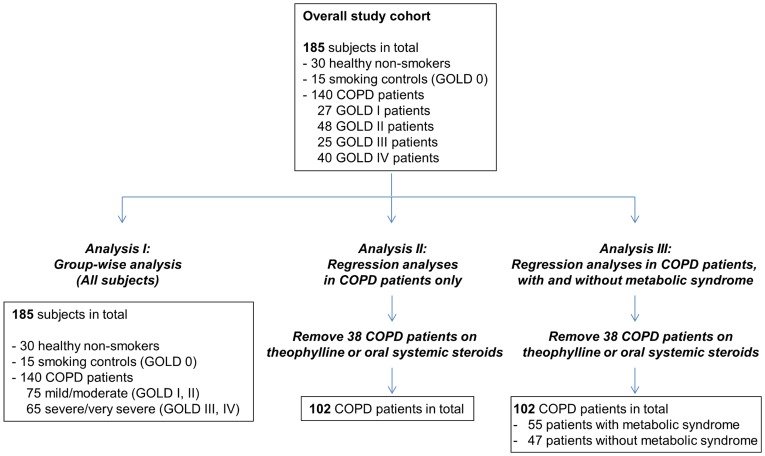
Overview of data analysis on COPD cohort.

We performed three sets of analyses on the biomarker data for this cohort (Figure 1). In the first analysis (analysis I or group-wise analysis), protein analyte levels were compared between mild/moderate COPD, severe/very severe COPD and control subject groups to identify biomarker differences between COPD and control subjects. Analysis I was performed on all 185 subjects in the cohort. In the second analysis (analysis II or regression analysis), biomarker levels were tested for correlation with lung function parameters on COPD patients only. To prevent possible confounding effects of pre-medication, analysis II was restricted to COPD patients not on theophylline or oral systemic steroids. Analysis II was thus performed on a smaller subset of 102 COPD patients. In the third analysis (analysis III), we asked if the associations of biomarkers with lung function parameters identified in analysis II were affected by the presence of coexisting metabolic syndrome. We divided the 102 COPD patients in analysis II into patients with (n  = 55) and without (n  = 47) metabolic syndrome and performed univariate correlation analysis in each patient subpopulation.

### Analysis I: Group-wise Analysis

**Figure 2:**
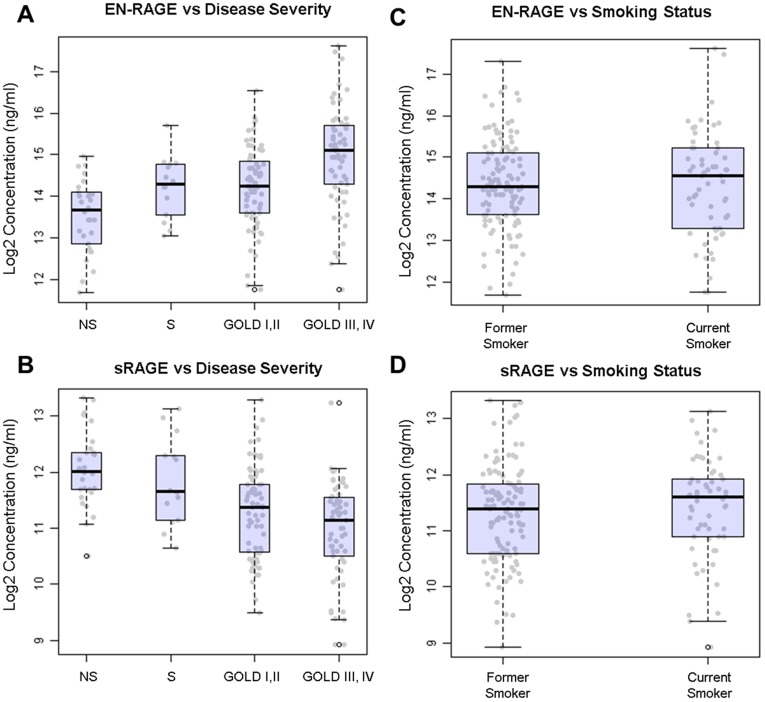
Increased serum EN-RAGE and decreased serum sRAGE are associated with COPD disease severity. Log2-transformed levels of EN-RAGE (A, C) and sRAGE (B, D) (ng/ml) for each subject are plotted with box-whisker plots illustrating median, 25^th^ and 75^th^ percentiles for non-smoking controls (NS), smoking controls (S), GOLD I and II (mild/moderate COPD) and GOLD III and IV (severe/very severe COPD) groups (A, B) and former and current smoker groups (C, D). Whiskers extend to data outside 1.5 times interquartile range.

Seven protein analytes showed significant differences across COPD disease severity groups (Table 2, Figure 2A and B, analysis details shown in Figure S1). Subjects were grouped as mild/moderate COPD (GOLD I, II, *n  = *75), severe/very severe COPD (GOLD III, IV, *n*  = 65), non-smoking (*n  = *30) and smoking (*n*  = 15) controls. Extracellular newly identified RAGE-binding protein (EN-RAGE; also known as S100A12), NGAL, Fibrinogen, transforming growth factor alpha (TGF-α), heparin-binding EGF-like growth factor (HB-EGF) and MPO were increased in COPD patients compared to controls, while soluble receptor for advanced glycation end products (sRAGE) was decreased in COPD patients. Only sRAGE and EN-RAGE displayed maximum pairwise fold-change differences in serum concentration greater than 1.5 fold; sRAGE decreased by 1.6-fold in severe/very severe COPD subjects compared to smoking controls, while EN-RAGE increased by 1.6-fold in subjects with severe/very severe COPD compared to mild/moderate COPD (Table S2). The trend for modest fold changes (<2 fold) persisted when analytes were compared across patients grouped by FEV_1_ quartile (Table S3). To confirm that these differences were not due to smoking status, we compared serum EN-RAGE and sRAGE levels in former and current smokers across the smoker, mild/moderate COPD and severe/very severe COPD groups and saw no differences in the levels of these analytes between these 2 groups (Figure 2C and D, *p*  = 0.52 for sRAGE, *p*  = 0.98 for EN-RAGE).

**Table 2:** Protein analyte differences between COPD and control disease severity groups.

Analyte	Non-Smokers (N = 30)	Smokers (N = 15)	GOLD I/II (N = 75)	GOLD III/IV (N = 65)	*P* (FDR)
EN-RAGE	13.1 (7.7 − 17.4)	20.0 (13.0 − 27.6)	19.5 (12.5 − 29.2)	35.5 (20.0 − 53.5)	0.001
TGF−α*	0.051 (0.045 − 0.055)	0.068 (0.053 − 0.075)	0.048 (0.042 − 0.060)	0.058 (0.045 − 0.073)	0.003
sRAGE	4.2 (3.3 − 5.2)	3.2 (2.4 − 5.0)	2.7 (1.5 − 3.5)	2.2 (1.5 − 3.0)	0.003
Fibrinogen	0.37 (0.34 − 0.42)	0.46 (0.44 − 0.49)	0.43 (0.37 − 0.47)	0.46 (0.42 − 0.53)	0.004
NGAL*	309.2 (258.6 − 354.2)	273.7 (233.2 − 338.1)	269.0 (211.0 − 340.2)	351.5 (272.5 − 441.5)	0.005
MPO*	414.5 (352.5 − 457.9)	382.0 (284.0 − 622.0)	379.0 (268.0 − 581.2)	553.0 (345.0 − 806.0)	0.02
HB-EGF*	0.18 (0.15 − 0.24)	0.23 (0.17 − 0.27)	0.18 (0.12 − 0.24)	0.27 (0.17 − 0.32)	0.02

Data are expressed as median (interquartile range) in ng/ml for individual analytes, except for Fibrinogen which is in mg/dl.

All analyte data shown are from profiling on the RBM Luminex platform, except for Fibrinogen which was tested at Hospital Grosshansdorf. COPD subjects were grouped as GOLD I/II (mild/moderate) and GOLD III/IV (severe/very severe). ANOVA was used for group-wise comparisons, except for analytes noted with *, which did not follow a normal distribution and a non-parametric Kruskal Wallis test was used.

Since the disease severity groups were unbalanced in terms of sample size, a post-hoc power analysis was also performed to assess the potential impact on statistical significance of the group-wise comparison results. The reduced number of subjects in the smoking control group affected the power to detect analytes with modest or weak effect sizes, especially after *p* value correction for multiple testing. EN-RAGE and NGAL, which displayed strong effect sizes (>0.25), were powered >0.6 at the α  = 0.0005 level, a stricter *p* value imposed by Bonferroni correction for multiple testing (Table S4).

### Analysis II: Regression Analysis in COPD Patients Only

Univariate regression was performed for each analyte against FEV_1%_predicted, FEV_1_/FVC or DLCO %predicted using data from 102 COPD subjects only (see Figure 1). COPD patients on theophylline and/or oral systemic steroids were excluded to avoid potential confounding effects of pre-medication. Four analytes were identified as significantly correlated with FEV_1_ including HB-EGF, EN-RAGE, TGF-α and MPO; 2 analytes with FEV_1_/FVC including HB-EGF and NGAL; and 9 analytes with DLCO including HB-EGF, TGF-α, monocyte chemotactic protein-4 (MCP-4), Sortilin, Fibrinogen, sRAGE, tissue inhibitor of metalloproteinase-1 (TIMP-1), VEGF and neutrophil-activating peptide-2 (NAP-2) (Table S5; |correlation| >0.3, *p*<0.05, FDR corrected for multiple testing). Interestingly, HB-EGF was the only analyte that was consistently correlated with FEV_1_, FEV_1_/FVC and DLCO, suggesting that it may be a general biomarker of lung function decline in COPD. With the exception of HB-EGF and TGF-α, analytes correlated with FEV_1_ were distinct from analytes correlated with DLCO. Moreover, whereas serum levels of EN-RAGE and sRAGE were both significantly different across COPD disease severity groups (Table 2 and Figure 2), regression analysis demonstrated that EN-RAGE correlated with FEV_1_ while sRAGE correlated with DLCO. A post-hoc power analysis showed that the sample size of the cohort was sufficiently powered to detect associations with correlation >0.35 with power >0.6 at the α  = 0.0005 level (Table S5). Analytes with stronger associations, such as NGAL with FEV_1_/FVC ratio and HB-EGF and MCP-4 with DLCO, were powered >0.8 at the α  = 0.0005 level.

Since univariate regression identified analytes that were modestly correlated with lung function parameters (|*r|*  = 0.3 to 0.45), we next performed multivariate regression to determine if the correlation with these same endpoints could be improved if a set of analytes were used as predictors instead of individual analytes. Multivariate analysis was performed using least angle regression (LARS) with 5-fold nested cross-validation (CV). The results are summarized in Table 3 with additional details in Table S6 and Methods S1. Notably, the predictor set for DLCO (HB-EGF, MCP-4, Fibrinogen, sRAGE and Sortilin) predicted a substantial percentage of COPD subject variability in the test set with a median value of 21%.

**Table 3:** Multivariate analysis of protein analyte data for COPD subjects.

Lung Function Parameter	Spearman Correlation	Adjusted R Squared	Analytes
FEV_1%_predicted	0.48	0.03	HB-EGF, EN-RAGE, MIP-1β
FEV_1_/FVC ratio	0.47	0.14	NGAL, HB-EGF, GST-α, MIP-1β
DLCO %predicted	0.43	0.21	HB-EBF, MCP-4, Fibrinogen, sRAGE, Sortilin

Spearman correlation and adjusted R squared values were computed using test set samples, in a 5-fold nested cross-validation scheme, averaged over 10 random seeds. R squared values were adjusted for the number of predictor terms in the model.

**Figure 3:**
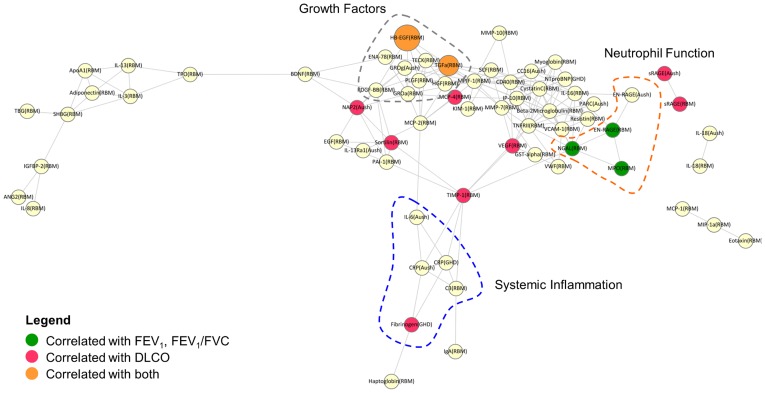
Correlation network illustrating functional co-clustering of analytes associated with FEV_1_, FEV_1_/FVC and DLCO. Analytes are plotted in a network using Cytoscape [83] where nodes represent analytes and edges represent significant correlations (*r* >0.4, *p*<0.05, corrected for multiple testing). Analytes are colored according to whether they were associated with FEV_1_ related parameters (green), DLCO (red) or both DLCO and FEV_1_ related parameters (orange) in univariate regression. Node size is proportional to the number of lung function parameters that showed significant association with a given analyte. Clusters of co-expressed analytes with similar function are highlighted by dotted regions in the graph as neutrophil function (orange), systemic inflammation (blue) and growth factor pathways (grey).

To better understand the functional association of the analytes predictive of FEV_1_, FEV_1_/FVC and DLCO in regression analyses, we asked if any of these proteins were enriched for common functions or were associated with common cellular pathways. Since proteins involved in similar functions and processes are likely to be correlated, our approach was to cluster the analytes selected in any one CV run from the multivariate analysis by correlation, and determine whether members of these clusters were enriched for association with specific lung function parameters assessed in the regression analyses. A correlation network (Figure 3) was constructed and three predominant clusters of analytes were identified. As expected, analytes in the same cluster were enriched for similar functions. For example, proteins associated with neutrophil function (EN-RAGE, NGAL and MPO) formed one cluster, while markers of systemic inflammation (IL-6, Fibrinogen, CRP) formed another cluster. A third cluster consisted of HB-EGF, TGF-α, platelet-derived growth factor (PDGF) and placental growth factor (PLGF), growth factors implicated in tissue injury and repair. When results from the univariate analysis (Table S5) were overlaid onto the network, we observed that members of the growth factor cluster (HB-EGF and TGF-α) were significantly correlated with both DLCO and FEV_1_ related parameters. In contrast, members of the neutrophil function cluster (EN-RAGE, NGAL, MPO) were uniformly associated with FEV_1_-related parameters but not DLCO. Apart from Fibrinogen, which was a member of the systemic inflammation cluster, analytes correlated with DLCO did not belong to any particular functional cluster, suggesting that whereas decline in FEV_1_ in COPD may be neutrophil associated, changes in DLCO may not be associated with any one specific cellular pathway or mechanism.

### Analysis III: Regression Analysis in COPD Patients with and Without Metabolic Syndrome

**Figure 4:**
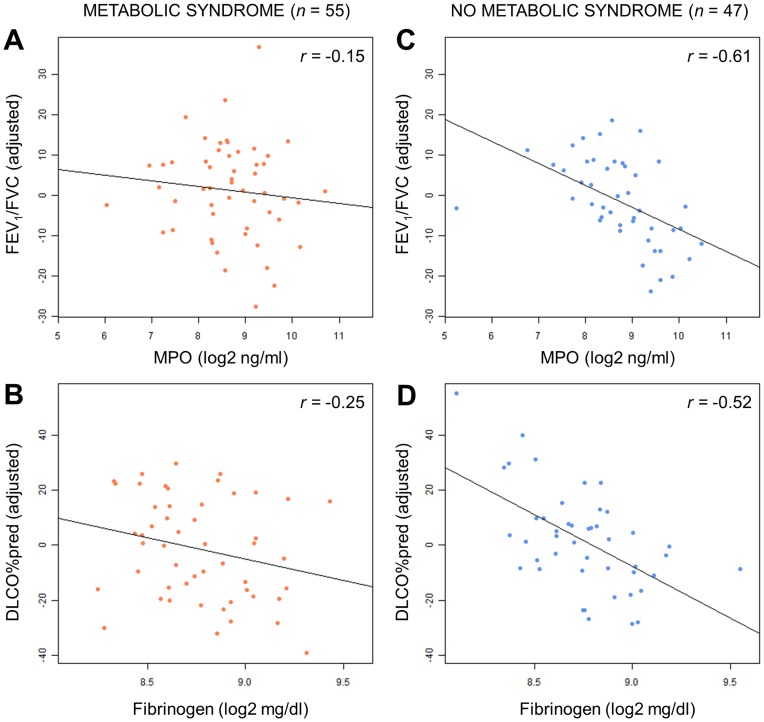
Association of MPO with FEV_1_/FVC and Fibrinogen with DLCO in COPD patients with and without metabolic syndrome. Log2-transformed levels of MPO (A, C) and Fibrinogen (B, D) (ng/ml for MPO and mg/dl for Fibrinogen) are plotted against covariate adjusted values for FEV_1_/FVC and DLCO, respectively in COPD patients with (A, B) and without (C, D) metabolic syndrome (*r* values indicate spearman correlation, covariates include age, sex, BMI, pack years and smoking status).

Next, we asked if biomarker associations with lung function parameters could be affected by the presence or absence of coexisting metabolic syndrome. To assess this, we first confirmed that COPD subjects with and without metabolic syndrome were well-matched and did not display any significant differences with respect to demographic covariates (Table S7). Analysis was performed on COPD patients not on theophylline and/or oral systemic steroids. COPD patients were subdivided based on the presence (*n*  = 55) or absence (*n*  = 47) of metabolic syndrome and univariate analysis was performed on each subpopulation (see Figure 1). MCP-4 was significantly correlated with DLCO in patients with metabolic syndrome, while Fibrinogen and HB-EGF were significantly associated with DLCO in patients without metabolic syndrome (Table 4). NGAL and MPO, both neutrophil associated markers, were also significantly associated with FEV_1_/FVC ratio in patients without metabolic syndrome. While the *p* value for interaction with metabolic syndrome was not significant at the *p*<0.05 level for any of these analytes, interaction *p* values for MPO and Fibrinogen were 0.09 and 0.08, respectively (Table 4). Both MPO and Fibrinogen were strongly negatively correlated with increasing FEV_1_/FVC (*r*  =  −0.61) and DLCO (*r*  =  −0.52), respectively, in patients without metabolic syndrome (Table 4 and Figure 4), suggesting that neutrophilia and systemic inflammation may be more strongly associated with disease severity in this subgroup of COPD patients. Multivariate analysis was attempted on COPD patients with and without metabolic syndrome, however, the sample size in these subgroups was too small to reliably perform cross-validation.

**Table 4:** Univariate regression analysis of protein analytes versus lung function parameters in COPD subjects with and without metabolic syndrome.

	With Metabolic Syndrome (*n* = 55)		Without Metabolic Syndrome (*n* = 47)		
Lung Function Parameter/Analyte	Spearman Correlation	*p* (FDR)	Spearman Correlation	*p* (FDR)	Interaction *p*
***FEV_1_/FVC ratio***					
MPO	−0.15	0.96	−0.61	0.023	0.09
NGAL	−0.35	0.25	−0.54	0.023	0.65
					
***DLCO % predicted***					
Fibrinogen	−0.25	0.47	−0.52	0.005	0.08
HB-EGF	−0.44	0.13	−0.45	0.027	0.55
MCP-4	−0.52	0.03	−0.28	0.342	0.29

Significance (*p* values) and effect sizes (spearman correlation) are listed for biomarker associations with lung function parameters. Interaction *p* values indicate significance of differences in biomarker associations with lung function parameters, between metabolic syndrome and non- metabolic syndrome groups.

## Discussion

In the present study, we performed a cross-sectional biomarker evaluation of samples from a COPD cohort, with the aim of using multiplex protein profiling to identify systemic biomarker changes associated with measures of disease severity in all COPD patients as a group, as well as in COPD patients with or without metabolic syndrome. Looking at all subjects, we identified 7 proteins with significantly different levels when comparing mild/moderate and severe/very severe COPD groups to smoking and non-smoking controls, as well as 12 proteins significantly correlated with at least one of the following lung function parameters in a univariate analysis: FEV_1_, FEV_1_/FVC and DLCO. In particular, using multivariate analysis we identified a 5-analyte predictor set comprised of HB-EGF, MCP-4, Fibrinogen, sRAGE and Sortilin that could explain up to 21% of subject variability in DLCO. When subjects were sub-grouped based on the presence of coexisting metabolic syndrome, we observed that MPO and Fibrinogen were strongly negatively associated with FEV_1_/FVC and DLCO, respectively, in patients without metabolic syndrome but not in patients with metabolic syndrome.

Our findings are consistent with previous biomarker studies in COPD, and extend these by relating known and novel markers to disease severity. For example, we demonstrated that Fibrinogen levels increase with COPD severity, consistent with its role as a marker of chronic inflammation and association with lung function decline in COPD [28], [29], [30]. MPO and NGAL, both neutrophil markers [14], [15], were increased with COPD disease severity and NGAL was correlated with FEV_1_/FVC by univariate and multivariate regression, consistent with the role of neutrophils in COPD pathogenesis [31] and recent reports that sputum neutrophilia increases with GOLD stage and is weakly associated with FEV_1_ [32]. Apart from its ability to reflect neutrophil activation, NGAL also functions as an antimicrobial and matrix degrading protein, the latter by binding to and inhibiting the inactivation of MMP-9 [14], suggesting a broader role for this marker in COPD. Indeed, NGAL has been proposed as a biomarker capable of predicting tissue injury in a number of acute and chronic inflammatory conditions such as vascular and renal disease [33].

One of the most striking findings in our study was the dichotomy of EN-RAGE and sRAGE association with COPD disease severity. When COPD patients were compared to smokers and non-smoking controls, EN-RAGE levels were significantly elevated in severe/very severe COPD patients, while sRAGE levels were significantly decreased with increasing disease severity. Univariate and multivariate regression further demonstrated that while EN-RAGE strongly associated with FEV_1_ and FEV_1_/FVC, sRAGE associated with DLCO, suggesting that these two members of the RAGE signaling pathway are differentially associated with measures of lung function decline and progression of lung destruction and emphysema.

RAGE is a multi-ligand receptor for diverse ligands such as advanced glycation end products (AGEs), S100 proteins (including S100A12/EN-RAGE) and high mobility group box 1 (HMGB1), and ligand-mediated activation of RAGE leads to inflammation and tissue injury [34], [35], [36], [37]. EN-RAGE is predominantly expressed by granulocytes and monocytes, and has a broad role in inflammatory responses [38], [39], [40], [41], [42], [43]. sRAGE is a soluble extracellular decoy receptor for RAGE that protects against inflammation and tissue injury [44], [45], [46]. In the lung, alveolar type I (AT-I) epithelial cells express high basal levels of RAGE [36] where it has been suggested to play an additional protective role by enhancing the interaction of AT-I cells with the basal lamina to promote effective gas exchange and alveolar stability [47]. In response to inflammation and lung injury [48], [49] sRAGE can be released from the alveolar epithelium via MMP-3 and MMP-13 dependent cleavage. RAGE ligands can also induce the expression of MMP-3 and MMP-13 [50]. Our observation of increased EN-RAGE and decreased sRAGE, with increased COPD severity suggests that the loss of sRAGE might be a consequence of alveolar epithelial cell loss due to MMP-induced matrix degradation and emphysematous lung destruction in severe COPD. The positive correlation on regression between decreased sRAGE and decreasing DLCO - a surrogate measure of emphysema - is consistent with this hypothesis. On the other hand, the correlation of increased EN-RAGE with a decline in FEV_1_ likely reflects high levels of underlying systemic inflammation in these patients, consistent with previous associations of CRP, IL-6 and Fibrinogen with COPD disease severity [7], [9], [29].

These findings support and extend an increasing number of studies demonstrating dysregulation of the RAGE pathway in chronic inflammatory disorders including arthritis, diabetes, and cardiovascular, peripheral vascular and respiratory diseases [35], [40], [48], [51], [52], [53]. In COPD, HMGB1, AGEs and RAGE have been shown to be overexpressed in the airways of COPD patients [54], [55]. Smith et al. [56] also recently showed that plasma sRAGE was reduced in patients with stable COPD and correlated with FEV_1_. A strong negative association of sRAGE with airway neutrophilia was also recently reported for asthma and COPD [57]. Several large genome-wide association studies also reported that single nucleotide polymorphisms in the AGER gene that codes for RAGE were associated with a reduced FEV_1_/FVC ratio indicative of airflow obstruction [58]. Taken together, these findings suggest that activation of the RAGE pathway may represent a nonspecific pathway of sustained inflammation and lung parenchymal tissue injury in COPD.

Another observation from this study was the strong association between HB-EGF and TGF-α with multiple lung function parameters in COPD. HB-EGF was the only analyte significantly associated with FEV_1_, FEV_1_/FVC and DLCO, suggesting that it may be a general marker of COPD disease severity. To our knowledge this is the first report of increased serum HB-EGF being associated with COPD disease severity. HB-EGF and TGF-α are ligands for the epidermal growth factor receptor (EGFR) that are synthesized as transmembrane precursors and subsequently released by proteolytic cleavage as biologically active mature proteins [59], [60]. HB-EGF and TGF-α have been shown to participate in epithelial repair processes and airway remodeling both *in vitro* and *in vivo* in models of lung disease [61], [62], [63], [64]. In particular, TGF-α has been associated with altered lung remodeling in human diseases such as chronic bronchopulmonary dysplasia and idiopathic pulmonary fibrosis (IPF) [65], [66], [67]. Increased protease activity and matrix breakdown during emphysema pathogenesis could increase HB-EGF and TGF-α levels in serum and account for the association of these proteins with lung function decline as measured by FEV_1_ and DLCO. EGFR and its ligands are normally weakly expressed in healthy airway epithelium but are upregulated in bronchial tissue of COPD patients by exposure to oxidative stress [68]. EGFR pathway activation can also be induced by exposure to oxidative stress, activated neutrophil supernatant or cigarette smoke [69], [70]. In turn, EGFR pathway activation can induce mucin production in airway epithelial cells and contribute to the mucus hyperproduction phenotype seen in COPD [69], [71]. These findings suggest a role for neutrophils in EGFR pathway activation that could explain the association of TGF-α and HB-EGF with disease severity and FEV_1_ in the current study. These findings also support the emerging idea that targeting lung repair mechanisms may be an approach for therapeutic intervention in COPD [72].

Comparing the results of the group-wise and regression analyses (i.e. analysis I and II), it is interesting that while both analyses identified similar analytes, the overlap between identified analytes was not 100%. This could be due to a number of reasons. First, subjects were divided into discrete groups in analysis I based on GOLD stage, which used specified cutoffs primarily based on FEV_1_, whereas in analysis II, FEV_1_ was used as a continuous variable for univariate regression with biomarker levels. Second, the power of group-wise tests (ANOVA or Kruskal-Wallis) to detect significant differences is sensitive to the number of samples in each group, whereas significance in univariate regression is dependent on the total number of samples. Third, only COPD patients were considered in univariate regression, whereas COPD patients were compared against non-COPD subjects in the group-wise analysis. Despite these differences, both methods identified analytes associated with epithelial repair (HB-EGF and TGF-α) and neutrophil function (EN-RAGE, MPO and NGAL) as significant, the latter being consistent with the hypothesis that neutrophil levels are strongly associated with the development of COPD, increasing COPD severity and FEV_1_ decline.

Using multivariate methods, we identified HB-EGF, MCP-4, Fibrinogen, sRAGE and Sortilin as a 5 analyte predictor set that could predict approximately 20% of test set patient variability in DLCO (adjusted R squared). This is significant given that few surrogate markers of DLCO have been reported in the COPD literature to date, and also because this evaluation of predictability was obtained on the test set in a cross-validation framework. These markers are associated with processes such as epithelial injury (sRAGE) and tissue repair (HB-EGF), consistent with the concept that DLCO is a surrogate marker of emphysema severity. Interestingly, markers of neutrophil function (i.e. NGAL and EN-RAGE) were associated with FEV_1_-related parameters but not DLCO.

Metabolic syndrome has been observed to be a frequent comorbidity in patients with COPD [73]. Patients with metabolic syndrome often present with higher levels of systemic inflammatory markers such as CRP, TNF-α, Fibrinogen and IL-6 [74], and these markers are also increased in the blood of COPD patients [7], [10]. In a prior study in our patient cohort [21], Watz et al. reported that the presence of metabolic syndrome was an independent predictor of IL-6 and hsCRP levels in COPD patients, with the levels of these markers displaying the same increasing trend with disease severity in COPD patients with and without metabolic syndrome, but being consistently higher in patients with coexisting metabolic syndrome. In the current study, CRP and IL-6 levels measured three years later in the same cohort were also higher in patients with metabolic syndrome but did not pass cutoffs for statistical significance (*p<*0.08 for CRP and *p*<0.13 for IL-6). Nonetheless, these findings suggest that COPD patients with metabolic syndrome face an added systemic inflammatory burden compared to COPD patients without metabolic syndrome, which could predispose them to worsened health outcomes and other comorbid diseases [23].

The independent contribution of metabolic syndrome to the increase in systemic levels of inflammatory markers may present an additional complication for biomarker studies. In our study, Fibrinogen and MPO displayed different associations with lung function parameters in COPD patients with and without coexisting metabolic syndrome, with Fibrinogen and MPO levels being significantly negatively correlated with DLCO and FEV_1_, respectively, only in patients without metabolic syndrome. Interestingly, both Fibrinogen and MPO were associated with DLCO and FEV_1_ when all patients were considered regardless of metabolic syndrome, suggesting that in the general population these associations may be driven by stronger associations within the non-metabolic syndrome subpopulation. Based on these findings, it may be useful to consider the presence of metabolic syndrome in future biomarker studies investigating the underlying molecular processes driving COPD pathogenesis.

There are a number of limitations to the current study that should be considered. This study was performed as a cross-sectional evaluation and therefore the associations of biomarkers with lung function parameters are correlative and not predictive. Furthermore, sample sizes across disease severity groups were unbalanced which affected the power to detect associations with modest effect size. Despite these limitations, we were able to distinguish significant associations of known and novel systemic biomarkers with multiple lung function parameters. This was likely facilitated by the fact that our subjects were recruited from a single center. Independent confirmation in larger cohorts across multiple centers, or longitudinal sampling studies will be needed to validate the novelty of biomarker associations identified in the current study. Nevertheless, our observations support the idea that distinct biological processes such as systemic inflammation, neutrophil activation and epithelial tissue injury/repair are associated with COPD disease severity. These pathways may therefore be linked to COPD pathophysiology and have the potential for identification of novel targets and biomarkers to address the unmet medical needs in these patients.

## Materials and Methods

### Ethics Statement

This study was performed in conformity with requirements approved by the institutional review board of Hoffmann-La Roche Inc. This study was approved by the local ethics committee of Schleswig-Holstein and all participants gave written informed consent.

### Subjects in Overall Study Cohort

Subjects in this study were recruited as part of an ongoing prospective observational study at the Pulmonary Research Institute at Hospital Grosshansdorf, Germany to examine the role of extrapulmonary effects of COPD including systemic inflammation and metabolic syndrome on disease severity and disease progression [21], [22], [24], [25], [26], [27]. The present study is a cross-sectional analysis of the follow up visit with this COPD cohort in 2008/2009, and comprises 30 healthy non-smokers, 15 smoking controls with chronic bronchitis (GOLD 0 in former GOLD classification scheme), and 140 patients with stable COPD (GOLD stage I-IV). Cross-sectional analyses of the baseline visit and survival analysis have been published previously [21], [22], [24], [25].

### Clinical Characteristics

Post-bronchodilator spirometry (performed 15 minutes after administration of 400 µg salbutamol) and DLCO were measured according to current guidelines using established reference values [75], [76], [77], [78]. The metabolic syndrome was assessed according to the criteria of the International Diabetes Federation as follows: central obesity (waist circumference ≥94 cm for men and ≥80 cm for women) plus any two of the following four factors: 1) triglycerides ≥150 mg/dL or specific treatment for this lipid abnormality; 2) high-density lipoprotein cholesterol <40 mg/dL in males and <50 mg/dL in females, or specific treatment for this lipid abnormality; 3) systolic blood pressure ≥130 mmHg or diastolic blood pressure ≥85 mmHg, or treatment of previously diagnosed hypertension; 4) fasting plasma glucose ≥100 mg/dL or previously diagnosed type 2 diabetes [79].

### Sample Collection and Analysis

Serum and plasma were collected at the time of the study visit at Hospital Grosshansdorf. Blood was collected separately into Sarstedt monovette syringes (Sarstedt Inc., Newton, NC) containing aluminum silica as the coagulant for serum collection, or citrate as the anti-coagulant for plasma collection. For serum collection, blood was allowed to stand at room temperature for 30 min and then centrifuged at 4°C for 10 minutes at 3000 rpm. Serum was removed, aliquoted and stored at −20°C within 1 hour of blood sampling. Plasma samples were used for the measurement of Fibrinogen levels according to the modified method of Clauss [80]. Serum levels of 142 protein analytes were measured using two multiplex platforms: Luminex multi-analyte profiling at Rules Based Medicine (RBM, Austin, TX) and Searchlight at Aushon Biosystems (Bellerica, MA). The RBM panel comprised 126 proteins and the Aushon Searchlight panel comprised 27 proteins. The analytes run on the Aushon platform were selected to complement the RBM panel and included analytes for which assays were not available with RBM (e.g. CC-16). Eleven analytes were evaluated on both platforms (CRP, NT-Pro-BNP, sRAGE, EN-RAGE, IL-6, sIL-6R, IL-10, IL-13, IL-18, PARC, Thrombopoietin), however, in these cases, only data from RBM was used in the biomarker analysis. Table S1 lists all 142 analytes tested and the associated multiplex platform.

### Analysis I: Group-wise Comparisons Using all Subjects

The goal of this analysis was to determine if biomarker levels differed across non-smoking control, smoking control, mild/moderate COPD (GOLD I, II) and severe/very severe COPD (GOLD III, IV) subject groups. Subject inclusion/exclusion criteria: All 185 subjects were included in this analysis. Subject numbers in each group are as follows: non-smoking controls (*n*  = 30), smoking controls with chronic bronchitis (*n*  = 15), mild/moderate COPD (GOLD I/II, *n  = *75), severe/very severe COPD (GOLD III/IV, *n*  = 65). Biomarker inclusion/exclusion criteria: Of the original set of 142 analytes that were measured (see Table S1), 113 were considered for analysis, while 14 analytes were eliminated due to too many missing values (missing in >80% of all subjects) and 15 analytes were eliminated that had fewer than 10 subjects in each group with valid values. Full details of the group-wise analysis can be found in Figure S1.

Statistical analysis: The fraction of missing data samples was compared across disease severity groups to identify non-random trends in missing data distribution (Table S8). IL-6, Eotaxin-3 and IL-1Rα showed non-random distributions. For example, IL-6 levels were below the limit of detection for most non-smoking control subjects, while IL-6 was present at detectable levels in smoking, mild/moderate and severe/very severe COPD subjects. Log transformation improved normality for most analytes, however, following log transformation the distributions for 74 of the 113 analytes remained insufficiently normal for application of parametric methods. A non-parametric test (Kruskal-Wallis) was used for these 74 analytes, while ANOVA was used for the 39 normally-distributed analytes. Protein analyte levels were adjusted for age, sex, BMI, pack years and current smoking status. Analytes with *p*<0.05 (FDR corrected for multiple testing) were considered significant. Table S1 lists all 142 analytes, whether they were normally distributed, the number of missing values, the type of test performed, and whether a significant association was discovered. R (version 2.13.2) was used for analysis. G*Power 3.1 was used for power analysis [81], [82].

### Analysis II: Regression Analyses in COPD Patients Only

The goal of this analysis was to identify systemic biomarkers associated with lung function parameters within COPD patients only. Subject inclusion/exclusion criteria: Of the 140 COPD patients in this cohort, 38 patients on theophylline and/or oral systemic steroids were removed to avoid potential confounding effects of pre-medication. Analysis was thus performed on the 102 remaining COPD patients. Biomarker inclusion/exclusion criteria: The same set of 113 analytes evaluated in Analysis I was used for regression analysis.

Statistical analysis: In univariate regression, protein analytes significantly associated with lung function parameters were identified by ANCOVA with lung function parameter and serum/plasma concentration level as dependent and independent variables. Age, gender, body mass index (BMI), pack years and smoking status were included in the model as covariates. Lung function parameters tested were post-bronchodilation FEV_1%_predicted (FEV_1_), post-bronchodilation FEV_1_/FVC ratio (FEV_1_/FVC) and DLCO %predicted (Hb-corrected). Analytes were considered significantly correlated if the absolute value of the Spearman correlation coefficient >0.3 and *p*<0.05, FDR corrected for multiple testing. Multivariate analysis was also performed to identify a set of analytes that when tested in combination, could explain a significant percentage of the variability of lung function parameter measures across COPD subjects. A least angle regression (LARS) procedure and five-fold, stratified, nested cross-validation (CV) was used to assess the predictive performance of the resulting LARS model (details in Methods S1). Analytes selected in >50% CV runs were used as a final multivariate predictor set for each lung function parameter. R (version 2.13.2) was used for analysis. G*Power 3.1 was used for power analysis [81], [82]. For the correlation network analysis, a network was constructed using Cytoscape [83] where an edge linking a pair of nodes represented analytes with a pairwise correlation >0.4. Analytes were colored green, red or orange if they were significantly correlated with FEV_1_ related parameters (FEV_1%_predicted, FEV_1_/FVC), DLCO or both, respectively. Node sizes in the network were proportionate to the number of lung function parameters that showed significance for any given analyte.

### Analysis III: Regression Analyses in COPD Patients with and without Metabolic Syndrome

The goal of this analysis was to determine if the biomarker associations with lung function parameters differed between COPD patients with and without metabolic syndrome. Subject inclusion/exclusion criteria: Similar to analysis II, analysis III was performed on 102 COPD patients not taking theophylline and/or oral systemic steroids. This population was divided into 2 subpopulations: 55 COPD patients with metabolic syndrome and 47 patients without metabolic syndrome. Biomarker inclusion/exclusion criteria: The same set of 113 analytes used in Analysis II was used for this analysis. Statistical analysis: The same univariate regression methods as Analysis II were used to assess biomarker associations with lung function parameters in the metabolic syndrome and non-metabolic syndrome subpopulations.

## Supporting Information

Figure S1 **Analysis workflow for analysis I: group-wise comparisons.**
(TIFF)

Table S1 **Full table listing number of missing values, median (IQR) in each group, test employed (parametric vs. non-parametric) and group-wise comparison significance for all 142 analytes.**
(XLS)

Table S2 **Post-hoc pairwise comparisons for protein analytes with significant differences across disease severity groups.** Analytes shown above had significant p values (p<0.05) using ANOVA or Kruskal-Wallis (*) test in group-wise comparison after correction for multiple testing with FDR (Table 2). Pairwise comparisons were computed using Tukey HSD test. NS: Non-smoking controls, S: Smoking controls, GOLD I/II: mild/moderate COPD, GOLD III/IV: severe/very severe COPD. Fold changes highlighted in bold represent pairwise comparisons driving significance in overall group comparison.
(DOC)

Table S3 **Post-hoc pairwise comparisons for protein analytes with significant differences across FEV1 quartile groups.** Analytes shown above had significant p value (p<0.05) using ANOVA or Kruskal-Wallis (*) test after correction for multiple testing with FDR. Pairwise comparisons were computed using Tukey HSD test. Subjects were grouped by FEV1 quartile: 1st: 0–25%, 2nd: 25−50%,3rd: 50–75%, 4th: 75–100%.
(DOC)

Table S4 **Post-hoc power analysis for analysis I: group-wise comparisons.** Analyte values were adjusted for age, gender, BMI, pack years and current smoking status. NS: Non-smoking controls, S: Smoking controls, GOLD I/II: mild/moderate COPD, GOLD III/IV: severe/very severe COPD. Power was calculated for two cases: i) significance at the p<0.05 level without multiple testing correction, and ii) significance at the p<0.05, correcting for multiple testing using the Bonferroni method with 100 analytes. Analysis was performed assuming a one-way ANOVA model. Bold values indicate analytes with power >0.6 at the α  = 0.0005 level.
(DOC)

Table S5 **Univariate regression and post-hoc power analysis of protein analytes versus lung function parameters.** Power was calculated for two cases: i) significance at the *p*<0.05 level without multiple testing correction, and ii) significance at the *p*<0.05, correcting for multiple testing using the Bonferroni method with 100 analytes. Bold values indicate analytes with power >0.6 at the α  = 0.0005 level.
(DOC)

Table S6 **Multivariate analysis of protein analyte data for COPD subjects.** All analytes, unless indicated otherwise, were profiled using the RBM Luminex platform. *Profiled using the Aushon Searchlight platform; **Profiled at Hospital Grosshansdorf. Abbreviations in addition to those in text of manuscript: MIP-1β: macrophage inflammatory protein1 beta; PAP: Prostatic acid phosphatase; GST-α: glutathione S-transferase alpha; ENA-78: epithelial neutrophil-activating peptide-78. The numbers in parentheses indicate the frequency with which this particular analyte was selected in the cross-validation runs of the multivariate analysis. The analytes highlighted in bold are those selected in >50% of the CV runs and represent the analytes used as a final multivariate predictor set for the particular lung function parameter.
(DOC)

Table S7 Demographics for COPD subjects with and without metabolic syndrome. Data are expressed as the number of subjects (% of subjects), mean (SEM) or median (Interquartile range) for lung function parameters
(DOC)

Table S8 **Distribution of samples with missing data values across disease severity groups.** Data are reported as n (fraction of samples with missing data compared to all samples in disease severity group). C.V indicates coefficient of variation  =  standard deviation of missing data fraction/mean missing data fraction across severity groups. NS: Non-smoking controls, S: Smoking controls, GOLD I/II: mild/moderate COPD, GOLD III/IV: severe/very severe COPD.
(DOC)

Methods S1 **Details of serum analyte analysis, multivariate model learning and performance evaluation.**
(DOC)
