# Supplementary figures and images for: Systemic Biomarkers of Neutrophilic Inflammation, Tissue Injury and Repair in COPD Patients with Differing Levels of Disease Severity
Source: PLoS One. 2012 Jun 12;7(6):e38629. doi: 10.1371/journal.pone.0038629 (PMC3373533; doi:10.1371/journal.pone.0038629)

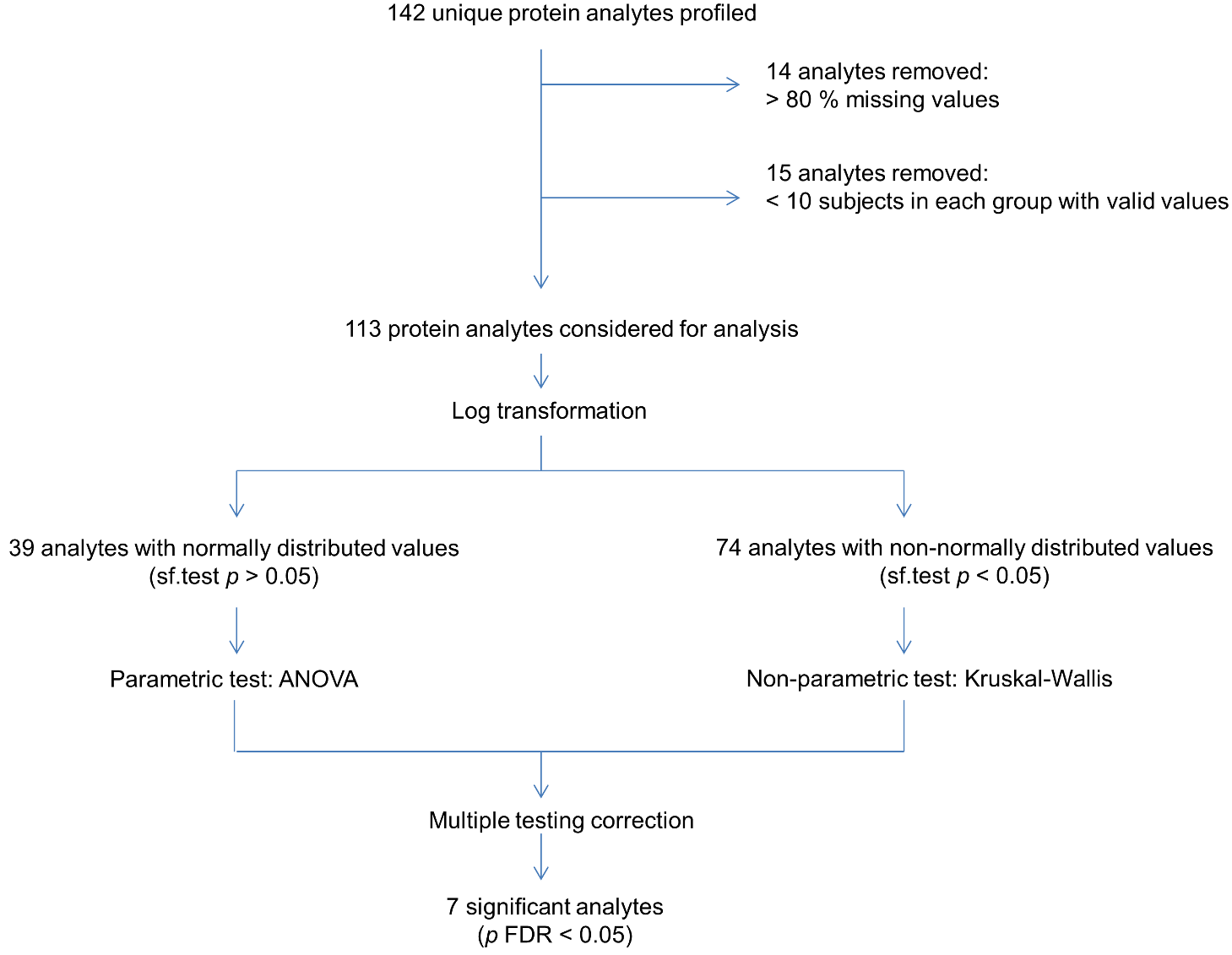

Supplement: Figure S1 — Analysis workflow for analysis I: group-wise comparisons. (TIFF) [file pone.0038629.s001.tiff]
