# Supplementary material for: Systemic Biomarkers of Neutrophilic Inflammation, Tissue Injury and Repair in COPD Patients with Differing Levels of Disease Severity
Source: PLoS One. 2012 Jun 12;7(6):e38629. doi: 10.1371/journal.pone.0038629 (PMC3373533; doi:10.1371/journal.pone.0038629)
Supplement: Table S2 — Post-hoc pairwise comparisons for protein analytes with significant differences across disease severity groups. Analytes shown above had significant p values (p<0.05) using ANOVA or Kruskal-Wallis (*) test in group-wise comparison after correction for multiple testing with FDR (Table 2). Pairwise comparisons were computed using Tukey HSD test. NS: Non-smoking controls, S: Smoking controls, GOLD I/II: mild/moderate COPD, GOLD III/IV: severe/very severe COPD. Fold changes highlighted in bold represent pairwise comparisons driving significance in overall group comparison. (DOC) [file pone.0038629.s003.doc]

**Supplementary Table 2.** Post-hoc pairwise comparisons for protein analytes with significant differences across disease severity groups.

| Analyte | *p*  (FDR) | Pairwise Fold-Changes | | | | | | Pairwise *p* Values | | | | | |
| --- | --- | --- | --- | --- | --- | --- | --- | --- | --- | --- | --- | --- | --- |
|  |  | S  vs. NS | GOLD I/II  vs. NS | GOLD III/IV  vs. NS | GOLD I/II  vs. S | GOLD III/IV  vs. S | GOLD I/II  vs. GOLD III/IV | S  vs. NS | GOLD I/II  vs. NS | GOLD III/IV  vs. NS | GOLD I/II  vs. S | GOLD III/IV  vs. S | GOLD I/II  vs. GOLD III/IV |
| EN-RAGE | 0.001 | 1.09 | -1.01 | 1.6 | -1.11 | 1.47 | **1.62** | 0.98 | 1 | 0.02 | 0.96 | 0.27 | < 0.001 |
| TGF-α* | 0.003 | 1.24 | -1.05 | 1.09 | **-1.3** | -1.14 | 1.15 | 0.46 | 0.96 | 0.82 | 0.19 | 0.78 | 0.3 |
| sRAGE | 0.003 | 1.18 | -1.13 | -1.37 | -1.33 | **-1.61** | -1.21 | 0.79 | 0.74 | 0.06 | 0.29 | 0.02 | 0.2 |
| Fibrinogen | 0.004 | 1.08 | -1.01 | 1.11 | -1.09 | 1.03 | **1.12** | 0.57 | 1 | 0.05 | 0.39 | 0.95 | < 0.001 |
| NGAL* | 0.005 | -1.12 | -1.18 | 1.16 | -1.05 | 1.3 | **1.37** | 0.81 | 0.23 | 0.33 | 0.98 | 0.11 | < 0.001 |
| MPO* | 0.02 | -1.13 | -1.18 | 1.22 | -1.04 | 1.39 | **1.44** | 0.91 | 0.57 | 0.43 | 1 | 0.25 | < 0.001 |
| HB-EGF* | 0.02 | 1.09 | -1.11 | 1.2 | -1.21 | 1.1 | **1.34** | 0.97 | 0.85 | 0.52 | 0.69 | 0.95 | 0.03 |

Analytes shown above had significant *p* values (*p* < 0.05) using ANOVA or Kruskal-Wallis (*) test in group-wise comparison after correction for multiple testing with FDR (Table 2). Pairwise comparisons were computed using Tukey HSD test. NS: Non-smoking controls, S: Smoking controls, GOLD I/II: mild/moderate COPD, GOLD III/IV: severe/very severe COPD. Fold changes highlighted in bold represent pairwise comparisons driving significance in overall group comparison.
