# Supplementary material for: Systemic Biomarkers of Neutrophilic Inflammation, Tissue Injury and Repair in COPD Patients with Differing Levels of Disease Severity
Source: PLoS One. 2012 Jun 12;7(6):e38629. doi: 10.1371/journal.pone.0038629 (PMC3373533; doi:10.1371/journal.pone.0038629)
Supplement: Table S3 — Post-hoc pairwise comparisons for protein analytes with significant differences across FEV1 quartile groups. Analytes shown above had significant p value (p<0.05) using ANOVA or Kruskal-Wallis (*) test after correction for multiple testing with FDR. Pairwise comparisons were computed using Tukey HSD test. Subjects were grouped by FEV1 quartile: 1st: 0–25%, 2nd: 25−50%,3rd: 50–75%, 4th: 75–100%. (DOC) [file pone.0038629.s004.doc]

**Supplementary Table 3.** Post-hoc pairwise comparisons for protein analytes with significant differences across FEV_1_ quartile groups.

| Analyte | *p*  (FDR) | Pairwise Fold-Changes | | | | | | Pairwise *p* values | | | | | |
| --- | --- | --- | --- | --- | --- | --- | --- | --- | --- | --- | --- | --- | --- |
|  |  | 2nd  vs. 1st | 3rd vs.1st | 4th  vs.1st | 3rd  vs. 2nd | 4th  vs. 2nd | 4th  vs. 3rd | 2nd  vs. 1st | 3rd vs.1st | 4th  vs.1st | 3rd  vs. 2nd | 4th  vs. 2nd | 4th  vs. 3rd |
| EN-RAGE | 0.002 | -1.44 | -1.75 | -1.73 | -1.21 | -1.2 | 1.01 | 0.07 | 0.002 | 0.002 | 0.58 | 0.61 | 1 |
| NGAL* | 0.008 | -1.18 | -1.5 | -1.25 | -1.27 | -1.06 | 1.2 | 0.2 | <0.001 | 0.04 | 0.02 | 0.9 | 0.14 |
| Fibrinogen | 0.009 | -1.08 | -1.16 | -1.11 | -1.07 | -1.02 | 1.05 | 0.15 | <0.001 | 0.04 | 0.24 | 0.94 | 0.56 |
| sRAGE | 0.009 | 1.1 | 1.21 | 1.45 | 1.1 | 1.31 | 1.19 | 0.83 | 0.35 | 0.01 | 0.85 | 0.1 | 0.44 |
| HB-EGF* | 0.01 | -1.04 | -1.51 | -1.09 | -1.46 | -1.05 | 1.39 | 0.99 | 0.01 | 0.9 | 0.02 | 0.98 | 0.05 |
| MPO* | 0.01 | -1.32 | -1.64 | -1.41 | -1.25 | -1.07 | 1.16 | 0.13 | <0.001 | 0.03 | 0.31 | 0.95 | 0.62 |
| PAP* | 0.02 | -1.34 | -1.4 | -1.21 | -1.04 | 1.11 | 1.16 | 0.003 | <0.001 | 0.11 | 0.96 | 0.6 | 0.31 |
| IL-15 | 0.05 | -1.04 | -1.11 | -1.24 | -1.07 | -1.2 | -1.12 | 0.97 | 0.64 | 0.06 | 0.88 | 0.17 | 0.57 |
| IL-1 α | 0.05 | -1.02 | -1.25 | -1.28 | -1.23 | -1.26 | -1.02 | 1 | 0.17 | 0.09 | 0.24 | 0.14 | 1 |
| TGF-α* | 0.05 | -1.02 | -1.25 | -1.01 | -1.23 | 1.01 | 1.23 | 1 | 0.1 | 1 | 0.16 | 1 | 0.13 |

Analytes shown above had significant *p* value (*p* < 0.05) using ANOVA or Kruskal-Wallis (*) test after correction for multiple testing with FDR. Pairwise comparisons were computed using Tukey HSD test. Subjects were grouped by FEV_1_ quartile: 1^st^: 0 – 25%, 2^nd^: 25 -50%,3^rd^: 50 – 75%, 4^th^: 75 – 100%.
