# Supplementary material for: Systemic Biomarkers of Neutrophilic Inflammation, Tissue Injury and Repair in COPD Patients with Differing Levels of Disease Severity
Source: PLoS One. 2012 Jun 12;7(6):e38629. doi: 10.1371/journal.pone.0038629 (PMC3373533; doi:10.1371/journal.pone.0038629)
Supplement: Table S4 — Post-hoc power analysis for analysis I: group-wise comparisons. Analyte values were adjusted for age, gender, BMI, pack years and current smoking status. NS: Non-smoking controls, S: Smoking controls, GOLD I/II: mild/moderate COPD, GOLD III/IV: severe/very severe COPD. Power was calculated for two cases: i) significance at the p<0.05 level without multiple testing correction, and ii) significance at the p<0.05, correcting for multiple testing using the Bonferroni method with 100 analytes. Analysis was performed assuming a one-way ANOVA model. Bold values indicate analytes with power >0.6 at the α = 0.0005 level. (DOC) [file pone.0038629.s005.doc]

**Supplementary Table 4.** Post-hoc power analysis for analysis I: group-wise comparisons

| Analyte | Covariate Adjusted Analyte Means | | | | SD | Effect Size | Non-Centrality parameter λ | Power | |
| --- | --- | --- | --- | --- | --- | --- | --- | --- | --- |
|  | NS  (*n* = 30) | S  *(n* = 15) | GOLD I/II  (*n* = 75) | GOLD III/IV  (*n* = 65) |  |  |  | α = 0.05 | α = 0.0005 |
| EN-RAGE | -0.23 | -0.11 | -0.25 | 0.45 | 1 | 0.32 | 19.6 | 0.97 | **0.64** |
| TGF-α | -0.04 | 0.27 | -0.11 | 0.09 | 0.6 | 0.20 | 7.2 | 0.59 | 0.10 |
| sRAGE | 0.21 | 0.45 | 0.03 | -0.24 | 0.8 | 0.23 | 9.55 | 0.85 | 0.32 |
| Fibrinogen | -0.05 | 0.05 | -0.06 | 0.09 | 0.3 | 0.23 | 10.1 | 0.75 | 0.20 |
| NGAL | 0.04 | -0.13 | -0.20 | 0.25 | 0.6 | 0.33 | 20.5 | 0.98 | **0.67** |
| MPO | 0.02 | -0.17 | -0.22 | 0.31 | 0.8 | 0.29 | 16.1 | 0.93 | 0.48 |
| HB-EGF | -0.04 | -0.09 | -0.19 | 0.23 | 0.8 | 0.23 | 9.8 | 0.74 | 0.19 |

Analyte values were adjusted for age, gender, BMI, pack years and current smoking status. NS: Non-smoking controls, S: Smoking controls, GOLD I/II: mild/moderate COPD, GOLD III/IV: severe/very severe COPD. Power was calculated for two cases: i) significance at the *p* < 0.05 level without multiple testing correction, and ii) significance at the *p* < 0.05, correcting for multiple testing using the Bonferroni method with 100 analytes. Analysis was performed assuming a one-way ANOVA model. Bold values indicate analytes with power > 0.6 at the α = 0.0005 level.
