# Supplementary material for: Systemic Biomarkers of Neutrophilic Inflammation, Tissue Injury and Repair in COPD Patients with Differing Levels of Disease Severity
Source: PLoS One. 2012 Jun 12;7(6):e38629. doi: 10.1371/journal.pone.0038629 (PMC3373533; doi:10.1371/journal.pone.0038629)
Supplement: Table S5 — Univariate regression and post-hoc power analysis of protein analytes versus lung function parameters. Power was calculated for two cases: i) significance at the p<0.05 level without multiple testing correction, and ii) significance at the p<0.05, correcting for multiple testing using the Bonferroni method with 100 analytes. Bold values indicate analytes with power >0.6 at the α = 0.0005 level. (DOC) [file pone.0038629.s006.doc]

**Supplementary Table 5.** Univariate regression and post-hoc power analysis of protein analytes versus lung function parameters.

| **Lung Function Parameter/Analyte** | **Spearman Correlation** | ***p* (FDR)** | **Power** | |
| --- | --- | --- | --- | --- |
|  |  |  | **α = 0.05** | **α = 0.0005** |
| ***FEV1 % predicted*** |  |  |  |  |
| HB-EGF | -0.36 | 0.024 | 0.98 | **0.65** |
| EN-RAGE | -0.37 | 0.024 | 0.99 | **0.7** |
| TGF-α | -0.35 | 0.024 | 0.97 | **0.61** |
| MPO | -0.35 | 0.041 | 0.97 | **0.61** |
| ***FEV1/FVC ratio*** |  |  |  |  |
| NGAL | -0.42 | 0.01 | 1 | **0.86** |
| HB-EGF | -0.34 | 0.02 | 0.97 | 0.57 |
| ***DLCO % predicted*** |  |  |  |  |
| HB-EGF | -0.45 | 0.0004 | 1 | **0.92** |
| TGF-α | -0.32 | 0.036 | 0.95 | 0.48 |
| MCP-4 | -0.42 | 0.003 | 1 | **0.86** |
| Sortilin | -0.36 | 0.003 | 0.98 | **0.65** |
| Fibrinogen | -0.37 | 0.003 | 0.99 | **0.7** |
| sRAGE | 0.37 | 0.004 | 0.99 | **0.7** |
| TIMP-1 | -0.31 | 0.018 | 0.93 | 0.43 |
| VEGF | -0.38 | 0.029 | 0.98 | **0.73** |
| NAP-2 | -0.31 | 0.029 | 0.93 | 0.43 |

Power was calculated for two cases: i) significance at the *p* < 0.05 level without multiple testing correction, and ii) significance at the *p* < 0.05, correcting for multiple testing using the Bonferroni method with 100 analytes. Bold values indicate analytes with power > 0.6 at the α = 0.0005 level.
