# Supplementary material for: Systemic Biomarkers of Neutrophilic Inflammation, Tissue Injury and Repair in COPD Patients with Differing Levels of Disease Severity
Source: PLoS One. 2012 Jun 12;7(6):e38629. doi: 10.1371/journal.pone.0038629 (PMC3373533; doi:10.1371/journal.pone.0038629)
Supplement: Table S6 — Multivariate analysis of protein analyte data for COPD subjects. All analytes, unless indicated otherwise, were profiled using the RBM Luminex platform. *Profiled using the Aushon Searchlight platform; **Profiled at Hospital Grosshansdorf. Abbreviations in addition to those in text of manuscript: MIP-1β: macrophage inflammatory protein1 beta; PAP: Prostatic acid phosphatase; GST-α: glutathione S-transferase alpha; ENA-78: epithelial neutrophil-activating peptide-78. The numbers in parentheses indicate the frequency with which this particular analyte was selected in the cross-validation runs of the multivariate analysis. The analytes highlighted in bold are those selected in >50% of the CV runs and represent the analytes used as a final multivariate predictor set for the particular lung function parameter. (DOC) [file pone.0038629.s007.doc]

**Supplementary Table 6: Multivariate analysis of protein analyte data for COPD subjects.**

|  |  | FEV_1_ %predicted | FEV_1_/FVC ratio | DLCO %predicted |
| --- | --- | --- | --- | --- |
| Test | Spearman Correlation Coefficient | 0.48 | 0.47 | 0.43 |
|  | Adjusted R Squared | 0.03 | 0.14 | 0.21 |
| Complete | Spearman Correlation Coefficient | 0.74 | 0.67 | 0.70 |
|  | Adjusted R Squared | 0.45 | 0.39 | 0.43 |
| Selected analytes (Frequency) | | **HB-EGF (86)** | **NGAL (90)** | **HB-EGF (97)** |
|  |  | **EN-RAGE^*^ (77)** | **HB-EGF (81)** | **MCP-4 (82)** |
|  |  | **MIP-1β (68)** | **GST-α (69)** | **Fibrinogen^**^ (79)** |
|  |  | MPO (48) | **MIP-1β (59)** | **sRAGE (68)** |
|  |  | TGF-α (48) | NAP2^*^ (41) | **Sortilin (66)** |
|  |  | MMP-10 (43) | PAP (32) | MMP-10 (33) |
|  |  | NGAL (41) | Fibrinogen^**^ (28) | PAP (32) |
|  |  | PAP (37) | TIMP-2^*^ (27) | VEGF (26) |
|  |  | Fibrinogen^**^  (32) | HMGB1^*^ (25) | ENA-78 (25) |
|  |  | IgM (27) | Haptoglobin (23) | sRAGE^*^ (14) |

All analytes, unless indicated otherwise, were profiled using the RBM Luminex platform. *Profiled using the Aushon Searchlight platform; **Profiled at Hospital Grosshansdorf. Abbreviations in addition to those in text of manuscript: MIP-1β: macrophage inflammatory protein1 beta; PAP: Prostatic acid phosphatase; GST-α: glutathione S-transferase alpha; ENA-78: epithelial neutrophil-activating peptide-78.

The numbers in parentheses indicate the frequency with which this particular analyte was selected in the cross-validation runs of the multivariate analysis. The analytes highlighted in bold are those selected in >50% of the CV runs and represent the analytes used as a final multivariate predictor set for the particular lung function parameter.
