# Supplementary material for: Systemic Biomarkers of Neutrophilic Inflammation, Tissue Injury and Repair in COPD Patients with Differing Levels of Disease Severity
Source: PLoS One. 2012 Jun 12;7(6):e38629. doi: 10.1371/journal.pone.0038629 (PMC3373533; doi:10.1371/journal.pone.0038629)
Supplement: Table S7 — Demographics for COPD subjects with and without metabolic syndrome. Data are expressed as the number of subjects (% of subjects), mean (SEM) or median (Interquartile range) for lung function parameters (DOC) [file pone.0038629.s008.doc]

**Supplementary Table 7:** Demographics for COPD subjects with and without metabolic syndrome.

|  | Without Metabolic Syndrome  (*n* = 47) | With Metabolic Syndrome  (*n* = 55) | *p* |
| --- | --- | --- | --- |
| ***Demographics*** | | | |
| Age in years | 66.7 (0.9) | 66.9 (0.9) | 0.92 |
| Males (*n*) | 33 (70.2) | 44 (80.0) | 0.26 |
| Body Mass Index (BMI) | 23.6 (0.6) | 28.7 (0.6) | < 0.001 |
| Pack years | 52.2 (3.5) | 59.8 (4.1) | 0.17 |
| Current smokers (*n*) | 23 (48.9) | 17 (30.9) | 0.06 |
| ***Lung*** ***function******parameters*** | | | |
| Post bronchodilator FEV_1_ % predicted | 58.0 (43.5 - 78.0) | 63.0 (50.5 - 82.5) | 0.34 |
| Post bronchodilator FEV_1_/ FVC ratio | 48.5 (37.5 - 61.2) | 55.4 (47.3 - 66.8) | 0.02 |
| DLCO % predicted (Hb corrected) | 52.6 (38.7 - 64.0) | 60.9 (43.3 - 71.5) | 0.27 |

| Data are expressed as the number of subjects (% of subjects), mean (SEM) or median (Interquartile range) for lung function parameters |
| --- |
