# Supplementary material for: Systemic Biomarkers of Neutrophilic Inflammation, Tissue Injury and Repair in COPD Patients with Differing Levels of Disease Severity
Source: PLoS One. 2012 Jun 12;7(6):e38629. doi: 10.1371/journal.pone.0038629 (PMC3373533; doi:10.1371/journal.pone.0038629)
Supplement: Table S8 — Distribution of samples with missing data values across disease severity groups. Data are reported as n (fraction of samples with missing data compared to all samples in disease severity group). C.V indicates coefficient of variation = standard deviation of missing data fraction/mean missing data fraction across severity groups. NS: Non-smoking controls, S: Smoking controls, GOLD I/II: mild/moderate COPD, GOLD III/IV: severe/very severe COPD. (DOC) [file pone.0038629.s009.doc]

**Supplementary Table 8.** Distribution of samples with missing data values across disease severity groups.

| Analyte | NS  (*n* = 30) | S  *(n* = 14) | GOLD I/II  (*n* = 75) | GOLD III/IV (*n* = 61) | C.V. |
| --- | --- | --- | --- | --- | --- |
| ***Analytes with non-random distribution of missing data across disease severity groups*** | | | | | |
| IL-6 | 26 (0.87) | 3 (0.21) | 26 (0.35) | 11 (0.18) | 0.79 |
| Eotaxin-3 | 16 (0.53) | 3 (0.21) | 20 (0.27) | 11 (0.18) | 0.54 |
| IL-1Rα | 3 (0.10) | 1 (0.07) | 3 (0.04) | 11 (0.18) | 0.61 |
| ***Analytes significant across disease severity groups by ANOVA*** | | | | | |
| EN-RAGE | 0 (0.00) | 0 (0.00) | 0 (0.00) | 0 (0.00) | 0 |
| NGAL | 0 (0.00) | 0 (0.00) | 0 (0.00) | 0 (0.00) | 0 |
| sRAGE | 0 (0.00) | 0 (0.00) | 0 (0.00) | 0 (0.00) | 0 |
| Fibrinogen | 0 (0.00) | 0 (0.00) | 0 (0.00) | 0 (0.00) | 0 |
| IL-10 | 1 (0.03) | 1 (0.07) | 0 (0.00) | 0 (0.00) | 1.3 |
| Tenascin-C | 0 (0.00) | 0 (0.00) | 0 (0.00) | 0 (0.00) | 0 |
| MPO | 0 (0.00) | 0 (0.00) | 0 (0.00) | 0 (0.00) | 0 |

Data are reported as *n* (fraction of samples with missing data compared to all samples in disease severity group). C.V indicates coefficient of variation = standard deviation of missing data fraction/mean missing data fraction across severity groups. NS: Non-smoking controls, S: Smoking controls, GOLD I/II: mild/moderate COPD, GOLD III/IV: severe/very severe COPD.
