# Supplementary material for: Systemic Biomarkers of Neutrophilic Inflammation, Tissue Injury and Repair in COPD Patients with Differing Levels of Disease Severity
Source: PLoS One. 2012 Jun 12;7(6):e38629. doi: 10.1371/journal.pone.0038629 (PMC3373533; doi:10.1371/journal.pone.0038629)
Supplement: Methods S1 — Details of serum analyte analysis, multivariate model learning and performance evaluation. (DOC) [file pone.0038629.s010.doc]

# Supplementary Methods S1:

## Serum analyte analysis

For analyte testing at RBM samples were shipped on dry ice. At RBM, each sample was thawed at room temperature, vortexed, spun at 13,000 x g for 5 minutes for clarification, and 40 µL was transferred into a master microtiter plate for MAP antigen analysis. An aliquot of each sample was introduced into one of the capture microsphere multiplexes of the Human Antigen MAP. The sample and capture microspheres were thoroughly mixed and incubated at room temperature for 1 hour. Multiplexed cocktails of biotinylated reporter antibodies for each multiplex were then added robotically and after thorough mixing, were incubated for an additional hour at room temperature. Multiplexes were developed using an excess of streptavidin-phycoerythrin solution mixed into each multiplex and incubated for 1 hour at room temperature. The volume of each multiplexed reaction was reduced by vacuum filtration and the volume increased by dilution into matrix buffer for analysis. Analysis was performed in a Luminex 100 instrument and the resulting data stream was interpreted using proprietary data analysis software. For each multiplex, both calibrators and controls were included on each microtiter plate. Eight-point calibrators were run in the first and last column of each plate and 3-level controls were included in duplicate. Testing results were determined first for the high, medium and low controls for each multiplex to ensure proper assay performance. All serum samples were run in duplicate and unknown values for each of the analytes localized in a specific multiplex were determined using 4 and 5 parameter, weighted and non-weighted curve fitting algorithms included in the data analysis package.

## Multivariate data analysis

For multivariate analysis, least angle regression (LARS) was used to identify a set of analytes that when tested in combination, explain a significant percentage of the variability of lung function parameter measures across COPD subjects. Five-fold, stratified, nested cross-validation (CV) was used to assess the predictive performance of the resulting LARS model. A nested cross-validation procedure was used where the algorithm iterated across two loops: an inner CV loop for model parameter optimization and an outer CV loop where model predictive performance was evaluated. The nested approach ensured that model parameters were not optimized using any of the data in the test set. In this procedure, the cohort was first divided into five outer splits in a stratified manner, where each split contained an even distribution of subjects by GOLD stage. One of the five outer splits was used as a validation set, while the remaining four splits were used as a training set. For the inner CV loop, each training set of four outer splits was further subdivided into five inner splits. LARS models were learned on four of the five inner splits as a function of increasing normalized shrinkage parameter *s* (ratio of L1 norm of the coefficient vector, relative to the norm at the full LS solution). *s* was allowed to vary from 0 to 0.2 in increments of 0.0025. Models were optimized for the *s* value yielding best performance on the remaining fifth inner test split. Performance was measured by the adjusted R squared value, which represented the percentage of variability explained, corrected by the number of variables in the model. Using the optimized *s* value obtained from iterating through the inner CV loop, a LARS model was learned using all data from the overall training set in the outer loop. The performance of this model was then evaluated on the validation set (fifth outer split). This procedure was repeated five-fold (using each outer split in turn as a validation set) for twenty random seeds, yielding a total of 100 CV runs. The median adjusted R squared and corresponding spearman correlation for the test set was reported, as well as a list of the most frequently selected features. Analytes selected in > 50% CV runs were used as a final multivariate predictor set for the particular lung function parameter.
